# Mimosa‐Inspired Body Temperature‐Responsive Shape Memory Polymer Networks: High Energy Densities and Multi‐Recyclability

**DOI:** 10.1002/advs.202407596

**Published:** 2024-08-14

**Authors:** Qingming Kong, Yu Tan, Haiyang Zhang, Tengyang Zhu, Yitan Li, Yongzheng Xing, Xu Wang

**Affiliations:** ^1^ National Engineering Research Center for Colloidal Materials School of Chemistry and Chemical Engineering Shandong University Jinan Shandong 250100 China

**Keywords:** body temperature responsiveness, dynamic covalent bonds, energy density, recyclable thermosetting materials, shape memory polymers

## Abstract

Inspired by the Mimosa plant, this study herein develops a unique dynamic shape memory polymer (SMP) network capable of transitioning from hard to pliable with heat, featuring reversible actuation, self‐healing, recyclability, and degradability. This material is adept at simulating the functionalities of artificial muscles for a variety of tasks, with a remarkable specific energy density of 1.8 J g^−1^—≈46 times higher than that of human skeletal muscle. As an intelligent manipulator, it demonstrates remarkable proficiency in identifying and handling items at high temperatures. Its suitable rate of shape recovery around human body temperature indicates its promising utility as an implant material for addressing acute obstructions. The dynamic covalent bonding within the network structure not only provides excellent resistance to solvents but also bestows remarkable abilities for self‐healing, reprocessing, and degradation. These attributes significantly boost its practicality and environmental sustainability. Anticipated to promote advancements in the sectors of biomedical devices, soft robotics, and smart actuators, this SMP network represents a forward leap in simulating artificial muscles, marking a stride toward the future of adaptive and sustainable technology.

## Graphical abstract

Inspired by the Mimosa plant, this study unveils a dynamic shape memory polymer network capable of transitioning from a rigid state to a flexible one near human body temperature. This innovative material offers smart functionalities, bolstered by high energy densities, and is characterized by its sustainable self‐healing properties, recyclability, and degradability, facilitated by dynamic covalent bonding.

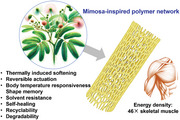

## 1 Introduction

Inspired by the dynamic adaptability of natural organisms, such as the rapid leaf movements of the Mimosa plant in response to touch, scientists have developed a range of shape memory polymers (SMPs) that replicate similar responsive behaviors.^[^ ^1^, ^2^, ^3^, ^4^, ^5^ ^]^ These polymers, capable of performing muscle‐like movements including expansion, contraction, and bending, along with more complex actions like intelligent locomotion and cargo handling, are categorized according to their responsiveness to temperature, electricity, and light.^[^ ^6^, ^7^, ^8^, ^9^, ^10^, ^11^ ^]^ Among these, thermotropic SMPs have been particularly noted for their excellent stability, reliability, and ease of control.^[^ ^12^, ^13^, ^14^, ^15^ ^]^ Prominent work led by Xie and colleagues revealed that thermotropic SMPs can undergo shape transformations at natural ambient temperatures, with their slow recovery speed opening up new possibilities for use in implantable medical devices.^[^ ^7^ ^]^ The capacity for these materials to perform mechanical work gently and gradually paves the way for complex procedures, resembling the nuanced movements of biomechanical systems. The triggering temperature and energy density of SMPs are critical for their practical applications in biomedicine and smart sensing. If the trigger temperature is too low, it will be difficult for the SMP to maintain its preset shape at room temperature. Conversely, excessively high trigger temperatures are unsuitable for human applications, as temperatures above 50 °C can cause irreversible tissue damage.^[^ ^16^ ^]^ Energy density is another important factor, as it measures the efficiency of SMPs and determines their capability for significant mechanical performance, such as functioning as artificial muscles with enhanced functionality.^[^ ^17^, ^18^ ^]^ Therefore, developing thermally responsive SMPs that operate at an ideal trigger temperature while maintaining high energy density is essential but remains a significant challenge. Moreover, as SMPs are increasingly utilized in varied and sometimes challenging environments, it becomes critical to ensure that their development and application are grounded in considerations of stability, recyclability, and environmental sustainability.^[^ ^19^, ^20^ ^]^

Thermoset SMPs, distinguished by their covalent network structure, are well‐known for their outstanding stability, chemical resistance, and superior mechanical attributes, which contribute to their prolonged operational life and heightened reliability.^[^ ^21^, ^22^, ^23^, ^24^ ^]^ However, the irreversible nature of covalent bonds within thermoset SMPs implies that any damage sustained during use permanently compromises the crosslinked network, adversely affecting the material's mechanical and functional properties, diminishing its lifespan, and potentially introducing safety risks.^[^ ^19^, ^25^ ^]^ Additionally, the impediments to recycling thermoset SMPs aggravate environmental degradation and the squandering of resources. The introduction of reversible covalent bonds overcomes these issues by amplifying the materials capacities for self‐repair, reusability, and eco‐friendly application.^[^ ^20^, ^26^, ^27^ ^]^ This advancement prolongs the materials service life, bolsters their reliability, and notably reduces cumulative maintenance costs, marking a significant step forward in sustainable materials science. Despite progress in responsiveness and reversibility, the endeavor to create SMPs that merge high energy density with mechanical and chemical robustness, alongside self‐healing, recyclability, and degradability, remains a significant challenge.

Drawing inspiration from the Mimosa plant, with its reversible actuation, self‐healing, and sustainable characteristics, we herein engineer a SMP network with a high energy density, responsiveness to body temperature, and multi‐recyclability, emulating the functionality of artificial muscles capable of reversible object transportation and selective gripping. Through the Schiff base reaction, polyetheramine is integrated into polyethylene glycol (PEG)‐based polyurethane to create dynamically imine crosslinked, recyclable, and degradable SMPs. Upon thermal activation, these newly synthesized SMP networks can contract or expand as needed, releasing energy during the recovery phase to either transport objects or alleviate obstructions in vessels. Furthermore, these polymer networks can facilitate the vertical lifting or falling of objects through the photothermal effect, or secure objects in a ring formation by means of transverse contraction. These SMP networks can mimic the function of an artificial arm for intelligent object handling, marking a significant advancement in the field of intelligent materials.

## 2 Results and Discussion

### 2.1 Synthesis and Characterization of SMP Networks

**Figure 1:**
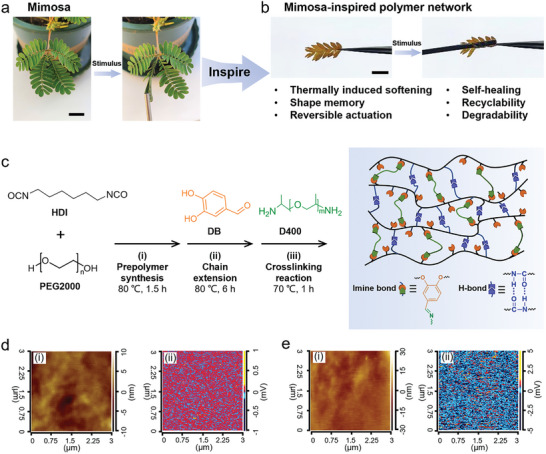
Visual comparisons and analytical imagery featuring the Mimosa and its synthetic counterpart. a) The Mimosa plant before and after tactile stimulation. Scale bar: 2 cm. b) The Mimosa‐inspired polymer network responding similarly. Scale bar: 1 cm. c) The synthesis pathway and the structural network of PBD_x_ polymers. d) AFM topography i) and AFM‐IR imagery at 1685 cm^−1^ ii) highlighting the localization of carbonyl (C═O) groups (indicated in red) in PBD_0_. e) AFM topography i) and AFM‐IR imagery at 1645 cm^−1^ ii) revealing the distribution of imine (C═N) groups (also in red) in PBD_0.5_.

The leaves of the Mimosa plant are diminutive and arranged in a pinnate fashion. They possess a unique responsiveness, contracting upon tactile stimulation due to an increase in the internal pressure within the leaf's vesicular cells.^[^ ^28^, ^29^ ^]^ This fascinating natural phenomenon inspired our development of a SMP network. This SMP network is characterized by reversible actuation capabilities and durability, mirroring the Mimosa's responsive action by curling up upon contact with a heated iron rod, as illustrated in **Figure** **1**a,b and Video S1 (Supporting Information). The synthesis process of these SMPs is depicted in Figure 1c. To create the SMP network, we commenced with the synthesis of an isocyanate‐terminated prepolymer through the polycondensation of PEG with a number‐average molecular weight (*M*_n_) of 2000 Da (PEG2000) and hexamethylene diisocyanate (HDI). Following this, a chain extender, 3,4‐dihydroxybenzaldehyde (DB), was incorporated to yield a linear polymer, designated as PBD_0_. The *M*_n_ of PBD_0_ was determined to be 30 343 Da by gel permeation chromatography (GPC), as shown in Figure S1 (Supporting Information), and its chemical structure was confirmed through proton nuclear magnetic resonance (^1^H NMR) analysis depicted in Figure S2 (Supporting Information). Subsequently, we introduced varying amounts of a crosslinking agent, poly(propylene glycol) bis(2‐aminopropyl ether) with a *M*_n_ of 400 Da (D400), into the mixture to synthesize a series of dynamic covalent crosslinking polymers, denoted as PBD_x_, with molar ratios (x) of the amino group in D400 to the aldehyde group in DB ranging from 0.25 to 1 (Table S1, Supporting Information).

Figure 1d presents the atomic force microscopy (AFM) topography i) and AFM‐infrared (AFM‐IR) absorption ii) images of PBD_0_, showcasing the distribution of C═O bonds at a specific wavelength of 1685 cm^−1^ in PBD_0_. This visual representation not only highlights the intricate surface characteristics of the polymer but also its chemical composition, providing a deeper insight into the polymer's structural properties. The AFM‐IR absorption image of PBD_0.5_, captured at a precise wavelength of 1645 cm^−1^, conclusively demonstrates the formation of C═N bonds (Figure 1e). This evidence underlines the successful incorporation of dynamic covalent bonds within the polymer structure, showcasing the intricate molecular engineering behind the development of these advanced materials.

**Figure 2:**
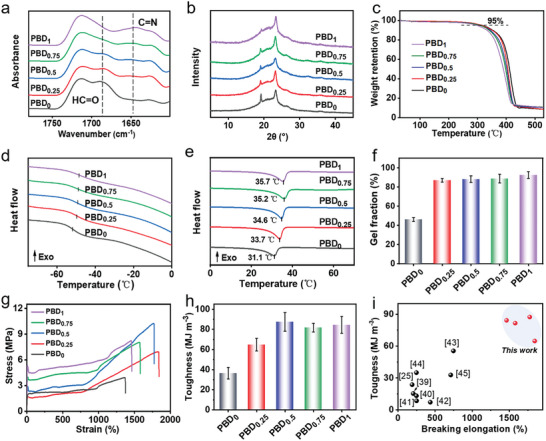
a) FTIR patterns, b) XRD curves, c) TGA curves, d,e) DSC curves with different temperature ranges, f) gel fraction, g) stress–strain curves, and h) toughness histograms of PBD_0_ and PBD_x_ (x = 0.25, 0.5, 0.75, and 1). i) Comparison of breaking elongation and toughness between PBD_x_ and other thermosetting polymers possessing shape memory and recyclability features, specifically focusing on those with fracture strains exceeding 100%.

Fourier transform infrared (FTIR) spectroscopy was utilized to examine the chemical composition of PBD_0_ (Figure S3, Supporting Information). The observed peaks at ≈1113 and 1685 cm^−1^ correspond to the ─C─O─C─ linkages in PEG2000 and the C═O bonds in urethane bonds, respectively.^[^ ^30^ ^]^ The notable absence of the stretching vibration peak of ─OH at 3333 cm^−1^ and the peak of ─NCO at 2250 cm^−1^ indicates the successful synthesis of PBD_0_.^[^ ^31^ ^]^ Furthermore, an increase in D400 content led to a gradual disappearance of the HC═O stretching vibration peak at 1685 cm^−1^, while the characteristic peak of C═N at 1645 cm^−1^ became increasingly pronounced (**Figure** **2**a). These FTIR findings corroborate the successful creation of PBD_x_ featuring a dynamic covalent network, which is consistent with the analysis of AFM‐IR. Complementary to this, X‐ray photoelectron spectroscopy (XPS) analysis of C 1s, N 1s, and O 1s spectra further validated the dynamic covalent network formation within PBD_x_ (Figures S4–S6, Supporting Information). X‐ray diffraction (XRD) analysis, detailed in Figure 2b, elucidated the crystal morphology of PBD_0_ and PBD_x_. PBD_0_ exhibited two distinct diffraction peaks at 19.2° and 23.2°, corresponding to the (120) and (032) crystal planes of PEG, respectively,^[^ ^32^, ^33^ ^]^ indicative of the polymer's potential for phase transition energy storage. With an increasing D400 ratio, the peak at 19.2° showed a gradual diminution, suggesting that the dynamic crosslinking structure lessens the regularity of the polymer chains.

Thermogravimetric analysis (TGA) demonstrates PBD_x_’s high thermal stability (Figure 2c). The differential scanning calorimetry (DSC) results, shown in Figure 2d,e, indicate a slight increase in both the glass transition temperature (*T*_g_) and phase transition point for crosslinked PBD_x_ compared to PBD_0_. This shift is attributed to the crosslinking points which limit molecular chain mobility, consequently reducing the average chain length between crosslinks and elevating both *T*_g_ and phase transition temperatures.^[^ ^34^, ^35^ ^]^ Gel content analysis, depicted in Figure 2f, further affirmed the crosslinked structure of PBD_x_. PBD_0_’s gel fraction stood at 44%, but significantly increased post‐crosslinking, signaling the formation of a robust crosslinked network. This comprehensive suite of analyses collectively confirms the intricate design and successful synthesis of PBD_x_ polymers with dynamic, covalently crosslinked structures, offering enhanced thermal stability and modified physical properties.

The mechanical attributes of the PBD_0_ and PBD_x_ series were investigated through stress–strain analyses (Figure 2g). When compared to the uncrosslinked PBD_0_, enhancements in both the stress at break and elongation at break were observed for the crosslinked PBD_x_ samples (Figure S7, Supporting Information). Notably, with a D400 content of 0.5, both the stress at break and toughness (the area surrounded by the stress–strain curve)^[^ ^36^ ^]^ attained their peaks, reaching 10.2 MPa and 87.4 MJ m^−3^, respectively (Figure 2g,h). The optimal quantity of crosslinking agents establishes dynamic covalent bonds within the polymer, enhancing the tensile strength and elongation at break of the polymer chains, thanks to these crosslinking sites.^[^ ^37^, ^38^ ^]^ Yet, when the internal network structure of the polymer grows too dense, it hampers the mobility of molecular chains, leading to a reduced stretching capacity. Consequently, PBD_0.5_ with a moderate crosslinking density was selected for subsequent experiments. It is important to highlight that, in comparison to recently reported shape memory thermoset polymers,^[^ ^25^, ^39^, ^40^, ^41^, ^42^, ^43^, ^44^, ^45^ ^]^ PBD_x_ exhibits considerable improvements in toughness (Figure 2i) and considerable adjustability in Young's modulus (Figure S8, Supporting Information). The enhanced stretchability and toughness of PBD_x_ stem from its dynamic crosslinking structure, which is adept at efficiently dissipating energy during stretching. The development of these SMPs focused on two key aspects: i) the chemical robustness of PEG, known for its resistance to oxidation and minimal environmental degradation, which secures the long‐term durability and dependability of PEG‐based SMPs; ii) reversible covalent bonds were incorporated into the polymer as crosslinking junctions, enhancing mechanical properties while preserving the polymer's processability. Consequently, these SMPs exhibit meticulously engineered toughness, stability, and recyclability, making them promising for widespread applications in artificial muscles, flexible actuators, and smart adaptive textiles.^[^ ^46^ ^]^

### 2.2 Thermally Induced Softening of PBD_0.5_

**Figure 3:**
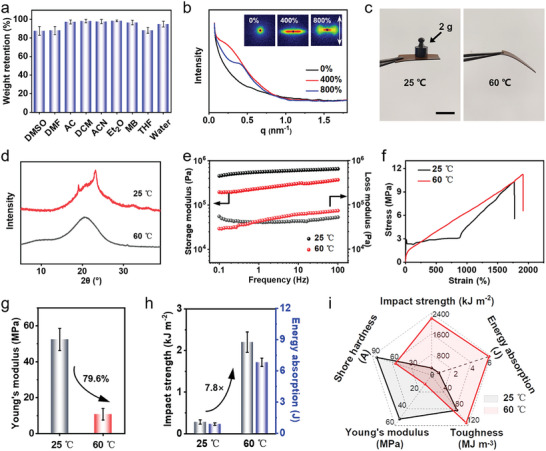
a) Weight retention of PBD_0.5_ soaked in different solvents for 24 h. The solvents include dimethyl sulfoxide (DMSO), *N*,*N*‐dimethylformamide (DMF), acetone (AC), dichloromethane (DCM), acetonitrile (ACN), ethyl ether (Et_2_O), methyl benzene (MB), tetrahydrofuran (THF), and water. b) SAXS patterns of PBD_0.5_ with different stretch ratios (0%, 400%, and 800%), c) A sample of PBD_0.5_, weighing 8.2 mg and measuring 22.8 mm × 10.2 mm × 0.032 mm, demonstrated the capability to support a 2 g weight at room temperature. However, it exhibited softening when exposed to a temperature of 60 °C. Scale bar: 1 cm. d) XRD curves of PBD_0.5_ at 25 and 60 °C. e) The dynamic rheological properties of PBD_0.5_ with frequency: the storage modulus (G′) and loss modulus (G″) versus frequency at 25 and 60 °C. f) Stress–strain curves and g) Young's moduli of PBD_0.5_ at 25 and 60 °C. h) Impact strength and energy absorption of PBD_0.5_ at 25 and 60 °C. i) Radar chart comparing Shore hardness, Young's modulus, toughness, impact strength, and energy absorption of PBD_0.5_ at 25 and 60 °C.

The solvent resistance of materials plays a pivotal role in fulfilling the requirements of diverse application contexts. According to **Figure** **3**a, it is evident that PBD_0.5_ exhibits robust solvent resistance across a range of common solvents, with the material retaining over 98% of its mass in dichloromethane (DCM). To delve into the nanoscale structural adaptations during the stretching of PBD_0.5_, in situ small‐angle X‐ray scattering (SAXS) analyses were performed (Figure 3b). Upon examining the sample at varied stretching ratios (400% and 800%), the dispersion rings intensified in the direction perpendicular to the stretching, leading to the formation of an ordered domain structure. At ambient temperature, the polymer's crystalline chains are immobilized, but external stress applied during stretching disrupts the crystal regions, prompting the emergence of micro‐nano crystalline zones. These zones align along the direction of tension, with the recrystallized regions expanding coherently with the stress orientation. As the extent of tension escalates, the smaller crystalline areas reorient to align with the stretching direction, culminating in a denser structural configuration. This reorientation is evidenced by the diffraction peak d‐spacing decreasing from 29.9 to 16.5 nm (stretching ratio from 400% to 800%), indicating a significant structural consolidation in response to mechanical stress. Furthermore, the radius of gyration (*R*_g_) can be obtained from the Guinier profile:^[^ ^47^ ^]^

(1) $$I\;\left(q\right)=a_{0}\;\exp\left(-\frac{R_{g}^{2}}{3}q^{2}\right)$$

where *q* is the scattering vector, *I*(*q*) is the scattering intensity, and *a*_0_ is a constant, to take the logarithm of both sides of this equation, we'd get:

(2) $$\ln\left[I\left(q\right)\right]\;=\;\ln a_{0}-\frac{R_{g}^{2}}{3}q^{2}$$

Thus, *R*_g_ can be derived from the slope of ln[*I*(*q*)] − *q* ^2^ curve in a small *q* region. It can be observed that *R*_g_ initially decreases and subsequently increases during tensile deformation (Figure S9, Supporting Information), effectively validating the previous hypothesis.

Interestingly, the PBD_0.5_ specimen was able to support a weight of 2 g at 25 °C, which is below its phase transition temperature, yet it exhibited significant softening when heated to 60 °C (Figure 3c). This temperature‐dependent behavior is essential for the material's shape memory capabilities.^[^ ^28^, ^48^ ^]^ As the temperature rises from 25 to 60 °C, the infrared data indicate that the absorption peak of the carbamate C═O group shifts from 1713 to 1718 cm^−1^, and the peak of the ether bond shifts from 1095 to 1100 cm^−1^ (Figure S10, Supporting Information). This suggests that hydrogen bonds play a crucial role in the shape memory process. To further understand the impact of temperature on the material structure, variable temperature XRD analysis was conducted (Figure 3d). At 60 °C, an increase in the width of the XRD peaks and a reduction in crystallinity were observed, indicative of the crystal phase melting and partial dissociation of hydrogen bonds at elevated temperatures, leading to diminished overall crystallinity.

Rheological analysis revealed that the storage modulus of the polymer decreases with rising temperature, suggesting the gradual mobilization of previously immobilized segments within the sample (Figure 3e). At ≈148 °C, the movement of polymer chains transitions from being restricted to unrestricted (Figure S11, Supporting Information). The dynamic mechanical thermal analysis (DMA) curve of PBD_0.5_ reveals two distinct peaks of loss factor, specifically 0.18 at −36.6 °C and 0.11 at 35.5 °C (Figure S12, Supporting Information). These findings align well with the DSC data (Figure 2d,e), indicating a phase transition in PBD_0.5_ ≈35.5 °C. Consequently, at room temperature (below the phase transition temperature), the sample undergoes plastic deformation during tension (Figure 3f). When the temperature remains below the phase transition threshold, the polymer chains are locked in place, resulting in plastic deformation under macroscopic tensile stress.^[^ ^49^ ^]^ However, as the temperature of the sample increases, the crystallized polymer chains thaw, and the hydrogen bond distribution is reorganized due to increased mobility of the polymer molecular segments, leading to elastic deformation on a macroscopic level.^[^ ^33^ ^]^

Upon elevating the test temperature to 60 °C, there was a 79.6% reduction in the Young's modulus. At the same time, this temperature rise significantly enhanced the impact strength and energy absorption of the material (Figure 3g,h). Comparisons of Shore hardness, Young's modulus, toughness, impact strength, and energy absorption of PBD_0.5_ at 25 and 60 °C (Figure 3i; Figure S13, Supporting Information) revealed that the material's chain segments are immobilized at room temperature but become mobilized upon heating, recovering elasticity. The distinct property transitions at different temperatures further confirm the change in the material's phase state, underscoring its potential as a shape memory material.

### 2.3 Applications of PBD_0.5_ in Shape Memory

**Figure 4:**
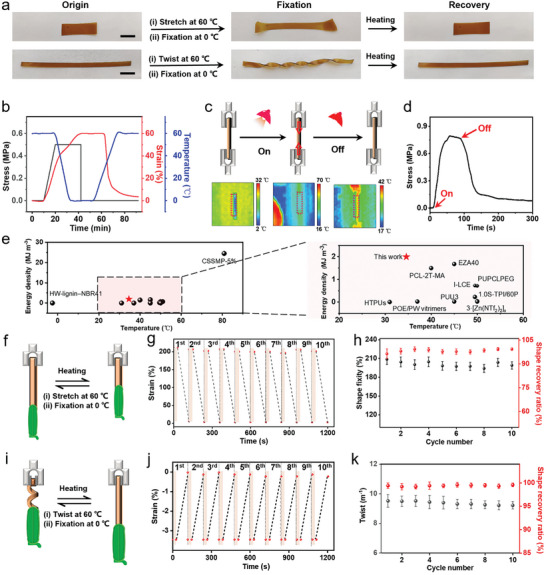
a) Shape memory processes (stretch and twist) of PBD_0.5_ during cyclic heating and cooling between 60 and 0 °C. Scale bars: 1 cm (the upper row) and 2 cm (the lower row). b) Dynamic mechanical curve of PBD_0.5_ under varying stresses of 0.6 and 0 MPa. c) Schematic representation and thermal imaging, and d) stress variations in the temporarily fixed sample at a strain of 500% on the universal tensile machine, before and after exposure to light. e) Comparison of temperature and volume energy density between PBD_0.5_ and other thermosetting polymers possessing shape memory and recyclability features (left) and the enlarged diagram at 20–60 °C (right). f) Schematic, g) initial strain and thermal recovery, and h) shape fixity and shape recovery ratio of the temporarily stretched sample lifting an object through thermal stimulation for ten cycles. i) Schematic, j) initial strain and thermal recovery, and k) twist and shape recovery ratio of the temporarily twisted sample releasing an object under thermal stimulation for ten cycles.

The sample possesses the ability to revert from temporary, programmed shapes back to its original form upon exposure to external thermal stimuli (Video S2, Supporting Information). **Figure** **4**a illustrates that PBD_0.5_ can exhibit various thermally induced shape memory behaviors, including stretching and twisting. To further elucidate the shape memory effect of the polymer, the entire process of shape programming and recovery was assessed using DMA (Figure 4b). Initially, heating the sample to 60 °C mobilized the polymer's chain segments. Under external stress, these molecular chains were stretched, yet the overall conformation of the polymer chains remained unchanged, thanks to the presence of crosslinking points. Cooling the sample under constant stress led to the re‐emergence of the oriented crystal phase and the reformation of the hydrogen bond network.^[^ ^46^ ^]^ Upon removal of the external stress, the sample's temporary shape was maintained due to restricted migration of macromolecules.^[^ ^50^ ^]^ When subsequently reheated to 60 °C, the internal stresses were alleviated, and the sample reverted to its initial shape.

To investigate the polymer's energy release under thermal stimulation, we measured the stress changes in samples with their tensile shape fixed at low temperature under the influence of photothermal effects. This process's change in shrinkage force was quantifiable using a stretching machine (Figure 4c). Specifically, the sample was elongated to 500% (the premeasured maximum recovery strain) of its original length and its shape was fixed at 0 °C. It was then mounted on a universal stretching machine and subjected to external thermal stimulation via a near‐infrared lamp. The curve in Figure 4d indicates that with a maximum recovery strain (*ε*_r,max_) of 500%, the maximum recoverable stress (*σ*_r,max_) reaches ≈0.8 MPa. This corresponds to an estimated volume energy density (*E* = 1/2*σ*_r,max_ *ε*_r,max_)^[^ ^17^, ^18^ ^]^ of 2.0 MJ m^−3^. The PBD_0.5_ material, with a density of ≈1.12 g cm^−3^, achieves an impressive specific energy density of 1.8 J g^−1^. This value is roughly 46 times higher than the energy density of human skeletal muscle, which is ≈0.039 J g^−1^.^[^ ^17^ ^]^ Compared to previously documented shape memory materials within the phase transition temperature range of 25–50 °C,^[^ ^51^, ^52^, ^53^, ^54^, ^55^, ^56^, ^57^, ^58^, ^59^, ^60^, ^61^ ^]^ our material, as illustrated in Figure 4e, exhibits a higher volume energy density. Moreover, it maintains this high energy density near human body temperature, making it particularly beneficial for applications in biomedicine and actuator development, where temperature responsiveness is crucial. The high energy density in our SMP network results from enhanced mechanical properties through optimal crosslinking, shown in Figure 2g, and the formation of ordered phase separation structures during stretching, depicted in Figure 3b. These features enable our material to efficiently store and release energy in response to external stimuli. The formation of dynamic covalent crosslinking and microphase separation structures enhances the entropy elasticity of the material, thereby endowing it with a high energy density. Turning off the lamp resulted in a gradual decrease in the shrinkage force, ultimately reaching equilibrium at 0.1 MPa. This decrease is attributed to the polymer's temperature gradually declining upon the lamp was off, leading to decreased activity in the chain and a reduction in contractile force. As the temperature stabilized back to room temperature, the sample's chain segments remained flexible, causing persistent internal stress and a certain level of shrinkage force at equilibrium.^[^ ^62^, ^63^ ^]^ The contraction force generated by the polymer during recovery could mimic artificial muscles, enabling the grasping of objects.^[^ ^64^, ^65^, ^66^ ^]^

Furthermore, we showcase the capability of PBD_0.5_ as an actuator, wherein the photothermal effect enables remote‐controlled manipulation of objects, encompassing both lifting and twisting actions. The shape memory effect's recovery and fixity ratios, *R*_r_ and *R*_f_, are determined using the equations below:^[^ ^15^ ^]^

(3) $$\;R_{r}=\frac{\left(\varepsilon -\varepsilon_{rec}\right)}{\varepsilon }\;\times 100\%$$

(4) $$\;R_{f}=\frac{\varepsilon }{\varepsilon_{load}}\;\times 100\%$$

where *ε* is the fixed strain after cooling and load removal, *ε*_rec_ is the strain after recovery, and *ε*_load_ represents the maximum strain under load. The twist, ∆*T*, of the sample is calculated from the following equation:^[^ ^67^ ^]^

(5) $$\Delta T\;=\frac{N\Delta L}{l^{2}}\;$$

where *N* represents the number of turns, *l* is length of the sample, and ∆*L* is the change in the length of the sample. Figure 4f shows the pre‐stretched PBD_0.5_ film (60 mg, dimensions 33.1 mm × 5.4 mm × 0.3 mm) successfully lifting a plastic clamp weighing 6.05 g upon heating with an infrared lamp (Video S3, Supporting Information). The specific work output (*W*, unit J g^−1^) can be calculated based on the following equation:^[^ ^17^ ^]^

(6) $$W\;=\frac{m_{1}}{m_{a}}\;gd$$

where *m*_l_ is the load mass, *m*_a_ is the actuator mass, *d* is the displacement, and *g* is the acceleration of gravity (9.8 m s^−2^). The polymer can lift more than 100 times its own weight, delivering a work output of 19.6 J kg^−1^. Maintaining elongation at ≈200%, the sample demonstrated a consistent shape memory effect over ten cycles (Figure 4g,h). Figure 4i–k depicts the pre‐twisted PBD_0.5_ film (0.13 g, dimensions 95.2 mm × 4.2 mm × 0.3 mm) gradually engaging with a plastic clamp weighing 6.05 g under continuous light exposure (Video S3, Supporting Information). The sample managed to lower the 6.05 g weight by 1.2 cm, equating to a work output of 5.5 J kg^−1^. Following ten cycles, the shape recovery rate maintained above 95%, indicating significant potential for use in reversible actuators.

**Figure 5:**
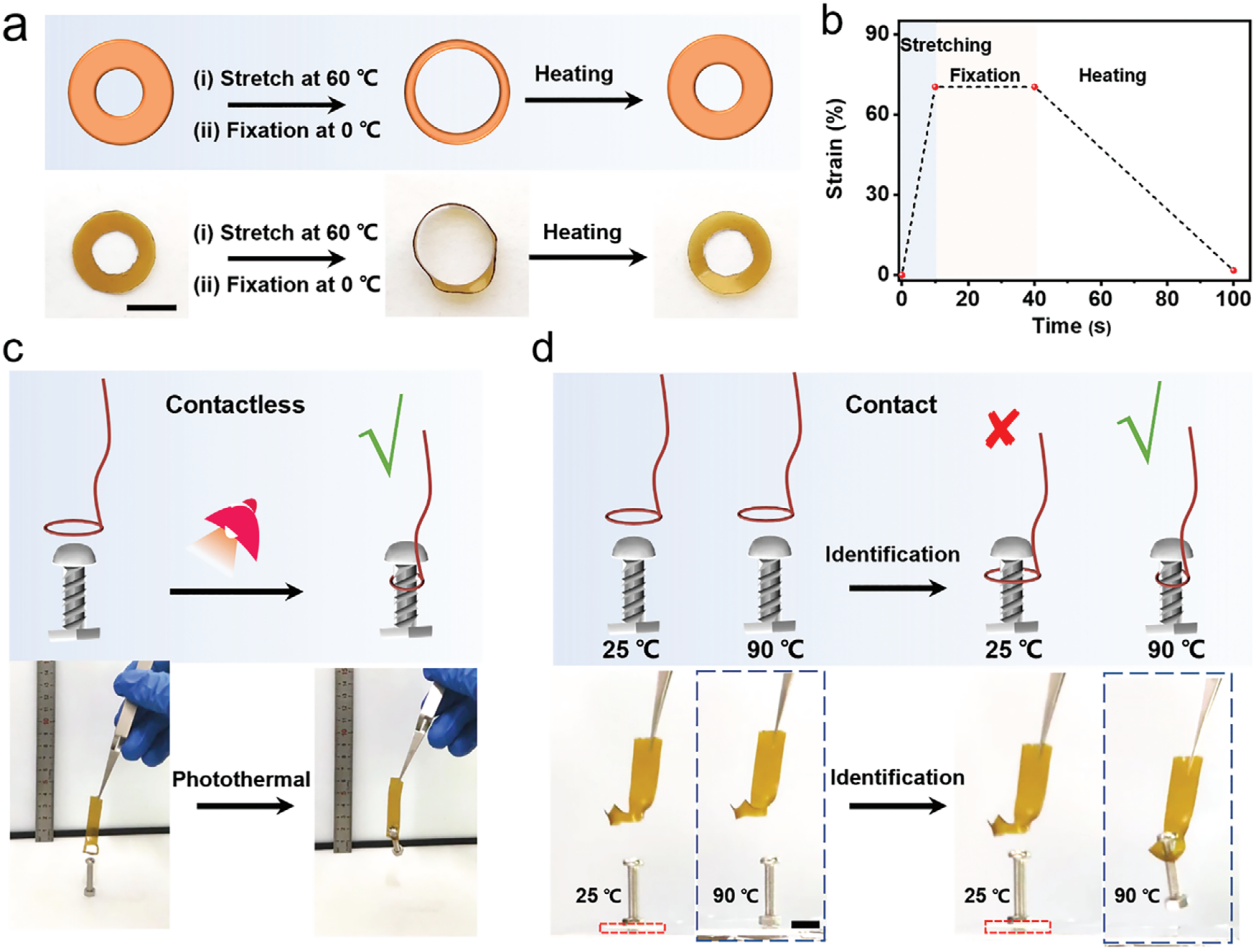
a) Schematic representation and actual photograph illustrating the shape memory behavior observed in annular samples. Scale bar: 1 cm. b) Changes in the diameter of the annular sample over time during the shape memory process. c) Schematic representation and actual photograph of the annular sample lifting an object without physical contact under infrared light illumination. d) Illustration of the selective gripping mechanism and an actual photograph showing the annular sample capturing a high‐temperature object. Scale bar: 1 cm.

Furthermore, the PBD_0.5_ sample can act as a thermally responsive artificial muscle, enabling the transport and selective gripping of objects. The ring‐shaped sample expanded and fixed its shape, followed by thermal stimulation that induced a contraction force, resulting in immediate shrinkage to grasp the object (**Figure** **5**a). Specifically, the inner diameter of the heat‐treated annular sample was increased to 1.7 times its original size, then immediately cooled at 0 °C for 30 s to fix its shape (Figure 5a,b). Subsequently, the sample was exposed to a temperature of 60 °C, where the ring's opening gradually recovered under thermal stimulation. One end of the sample was fashioned into a ring, and this fixed ring at low temperature was inserted around the object. Upon remote irradiation with infrared light, the ring began to contract, securing the object, thereby facilitating the contactless transport of objects, as illustrated in Figure 5c and Video S4 (Supporting Information).

Additionally, this mechanism serves as a smart gripper, selectively engaging objects that are at high temperatures. As demonstrated in Figure 5d, one of the objects was heated directly to 90 °C, while another was placed on heat‐insulating filter paper. When the pre‐expanded annular PBD_0.5_ sample came into contact with the objects, it retained the low‐temperature object on the filter paper, while effectively capturing the high‐temperature object (Video S5, Supporting Information). This capability to discern and intelligently manipulate objects holds significant promise for the advancement of flexible intelligent actuators.

**Figure 6:**
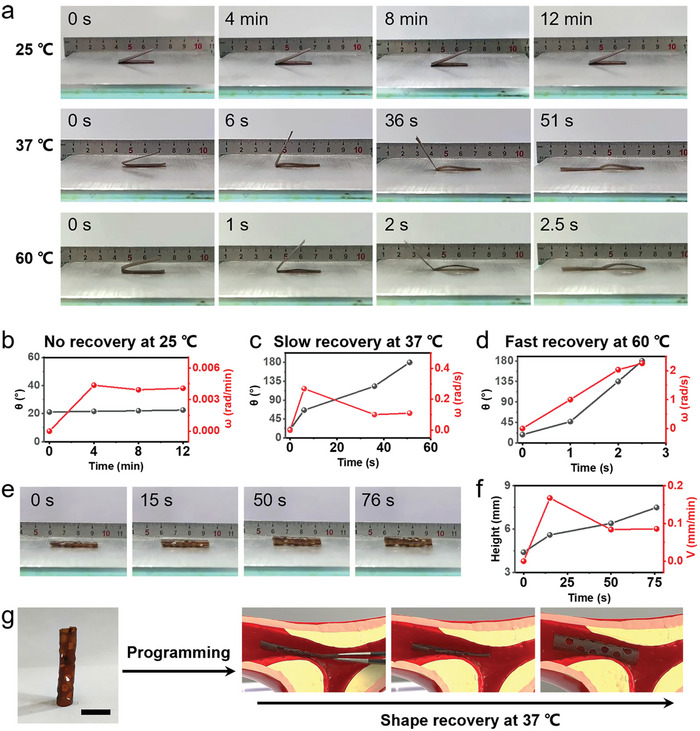
a) Visual demonstration of the shape recovery processes of PBD_0.5_ films at temperatures of 25, 37, and 60 °C. b–d) The correlations between recovery rate and time during the shape recovery processes of PBD_0.5_ films at (b) 25, (c) 37, and (d) 60 °C. e) Visual demonstration of the shape recovery process of a PBD_0.5_ stent at 37 °C. f) The correlation between recovery rate and time during the shape recovery process of a PBD_0.5_ stent at 37 °C. g) Photographs demonstrating the potential application of PBD_0.5_ in vascular stents. Scale bar: 1 cm.

The shape recovery rates of PBD_0.5_ under different temperatures were investigated. The sample was positioned on a heating plate set at temperatures of 25, 37, and 60 °C, respectively, during which both the shape change and shape recovery rate were meticulously documented (**Figure** **6**a). The sample film, initially set at a temporary angle of 21.1°, remained virtually unchanged after being placed on a heating plate at 25 °C (Video S6, Supporting Information). This occurred because the ambient room temperature falls below the phase transition temperature of PBD_0.5_. However, as the temperature increased to 37 and 60 °C, it underwent recovery in 51 and 2.5 s, respectively (Video S6, Supporting Information). Figure 6b illustrates that the PBD_0.5_ film hardly recovered at 25 °C. At 37 °C, a temperature marginally above PBD_0.5′_s phase transition point, the sample recovered slowly, with a final recovery rate (*ω* = Δ*θ/t*, where Δ*θ* is the angle after recovery at time *t*) of 0.11 rad s^−1^ (Figure 6c). As the temperature reached 60 °C, the recovery rate of the sample markedly accelerated, achieving a significant increase to 2.3 rad s^−1^ by the 2.5‐s mark (Figure 6d). Additionally, we evaluated the recovery behavior of temporarily stretched samples at temperatures of 25, 37, and 60 °C. The outcomes paralleled those observed with bending, showcasing a slow activation ≈37 °C (Figure S14 and Video S7, Supporting Information).

Given the slow recovery properties of our SMP network at temperatures close to that of the human body, it holds potential as an implant for addressing acute blockages, instantaneously expanding narrowed areas, and achieving hemostasis, among other applications.^[^ ^7^ ^]^ To explore this potential, we compressed a 3D hollow cylindrical sample under external force and cooled it to 0 °C, creating a temporarily flattened scaffold (Figure 6e). Subsequently, it was placed on a heating plate set at 37 °C, where it gradually reverted to its original shape, achieving a final recovery rate (*v* = Δ*l/t*, where Δ*l* is the strain after recovery at time *t*) of 0.09 mm min^−1^ (Figure 6f; Video S8, Supporting Information). This attribute was leveraged to insert the material into a blood vessel as a stent (Figure 6g), illustrating that its progressive expansion at 37 °C could be effectively utilized for medical stents.

### 2.4 Repairability, Weldability and Recyclability of PBD_0.5_

**Figure 7:**
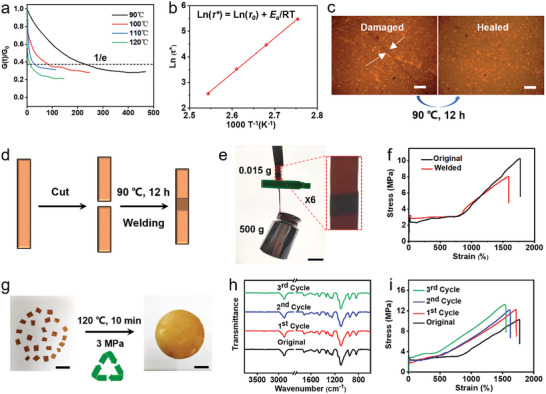
a) Stress relaxation curves for PBD_0.5_ at various temperatures. b) Activation energy for PBD_0.5_ determined by applying the Arrhenius equation to relaxation time. c) A digital photograph showing PBD_0.5_ before and after healing (at 90 °C for 12 h). Scale bar: 200 µm. d) Illustrative diagram of the sample welding process post‐cutting. e) Digital photographs of the welded PBD_0.5_ sample (15 mg) supporting a weight of 500 g. Scale bars: 2 cm. f) Stress–strain curves for PBD_0.5_ before damage and post‐welding. g) The hot‐pressing recovery process for PBD_0.5_. Scale bars: 2 cm (left) and 1 cm (right). h) FTIR spectra and i) stress–strain curves for PBD_0.5_ following three cycles of hot‐pressing recovery.

Given the dynamic and complex nature of the environment during practical applications of shape memory materials, aspects such as self‐healing, recyclability, and degradability of these materials are critically important for their advancement. Stress relaxation experiments conducted on the samples at various temperatures investigated the reversibility of the dynamic crosslinking network.^[^ ^68^ ^]^ As temperatures were raised, the characteristic relaxation time (τ*) gradually decreases (**Figure** **7**a). This decrease in τ* with rising temperature indicates accelerated chain movements within the sample, enhancing the exchange of dynamic imine bonds and leading to the topological rearrangement of the crosslinked network.^[^ ^45^ ^]^ A clear linear relationship was observed between ln(τ*) and 1/(1000T), with an activation energy (*E*_a_) of 114.7 kJ mol^−1^ (Figure 7b). The PBD_0.5_ sample exhibited Arrhenius flow characteristics, advantageous for self‐healing, weldability, and reprocessability. We also determined the topological freezing transition temperature (*T*_v_) of PBD_0.5_ by extrapolating from stress relaxation experiments and calculated *T*_v_ to be ≈31.2 °C.

To assess self‐healing capabilities, a PBD_0.5_ sample scratched with a blade was placed in an oven at 90 °C to heal for 12 h, resulting in almost complete recovery (Figure 7c). Additionally, two halves of a cut sample were overlapped and welded at 90 °C for 12 h (Figure 7d). The welded sample could lift objects ≈33 000 times its own weight, and its toughness and elongation at break were restored to 80.2% and 88.3%, respectively (Figure 7e,f).

The reprocessing of the sample was achieved through hot‐pressing: the material was cut into pieces and then subjected to hot‐pressing at 120 °C under a pressure of 3 MPa for 10 min, illustrating the material's reprocessability (Figure 7g). The FTIR spectra indicated no significant changes in the chemical structure of the samples after multiple reprocessing cycles, showcasing their excellent recoverability (Figure 7h). The stress–strain curves revealed that the mechanical properties of the samples could be almost fully restored after several reprocessing cycles (Figure 7i).

**Figure 8:**
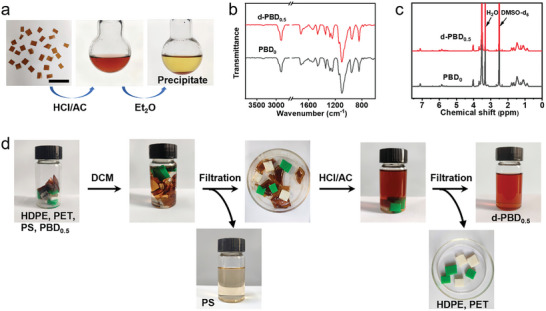
a) The degradation process of PBD_0.5_ illustrated. Scale bar: 2 cm. b) FTIR and c) ^1^H NMR spectra of the degradation products from PBD_0.5_, compared with PBD_0_. d) Images showing the selective separation process of PBD_0.5_ from a plastic waste mixture containing HDPE (green), PET (white), PS (colorless and transparent), and PBD_0.5_ (dark brown).

Furthermore, the sustainable development of the material can be facilitated through degradation when its performance diminishes. The dynamic imine crosslinked network within the sample is known to break down easily under acidic conditions.^[^ ^68^, ^69^ ^]^ At room temperature, a mixture consisting of acetone (AC) and an aqueous solution of hydrochloric acid (HCl) in a ratio of 9:1 (v/v) was introduced to a PBD_0.5_ sample, causing the sample block to gradually degrade and the solution to turn yellow (**Figure** **8**a). After continuous stirring for 6 h, the precipitate was separated using ethyl ether (Et_2_O), allowed to settle, then washed repeatedly with water and ether, and finally oven‐dried at 60 °C (yield of 82%). The degradation product (i.e., d‐PBD_0.5_), after recovery and purification, closely matched the FTIR and ^1^H NMR spectra of PBD_0_ (Figure 8b,c), indicating the sample's excellent degradability. This fact also highlights the promise of our polymer networks for closed‐loop recycling. In addition, PBD_0.5_ fragments were combined with three commercially available plastics: high‐density polyethylene (HDPE, characterized as green), poly(ethylene terephthalate) (PET, identified as white), and polystyrene (PS, which is colorless and transparent). These mixed plastics were submerged in DCM and subsequently filtered to isolate PS. The mixture was then introduced into a solution comprising AC and 1 m HCl to extract the recoverable form of d‐PBD_0.5_. Meanwhile, the remaining plastics, HDPE and PET, were left unaltered (Figure 8d). This observation suggests that our SMP network could potentially enable selective depolymerization and separation within a heterogeneous waste stream.

## 3 Conclusion

Inspired by the Mimosa plant, we develop a healable and recyclable SMP network capable of releasing stored energy upon thermal activation, facilitating muscle‐like contractile movements. Upon photothermal stimulation, the material demonstrates an energy output of 19.6 J kg^−1^ via stretching and 5.5 J kg^−1^ through curling, alongside a high specific energy density of 1.8 J g^−1^ when pre‐stretched. These characteristics offer significant advantages for contactless control of cargo transportations. This SMP network can also serve as an intelligent gripping mechanism, enabling the thermo‐specific identification and capturing of high‐temperature objects from a stack of samples. Additionally, its trigger response temperature is near human body temperature, and its slow recovery at ≈37 °C makes it a promising candidate for implantable materials aimed at resolving acute obstructions. The covalent crosslinking structure of the SMP network grants it outstanding solvent resistance and thermal stability, enhancing its suitability for challenging environments. The dynamic covalent bonds within the material support notable self‐healing, reprocessing, and degradation capabilities, contributing to the material's sustainability. The progress of our SMP network offers considerable potential to boost reliability and environmental sustainability in practical applications, opening up new opportunities for the innovation of smart materials.

## 4 Experimental Section

### Materials

Polyethylene glycol with a number average molecular weight of 2000 Da (PEG2000) and 3,4‐dihydroxybenzaldehyde (DB) were purchased from Shanghai Macklin Biochemical Technology Co., Ltd. Dibutyltin dilaurate (DBTDL) was purchased form Sigma–Aldrich. Hexamethylene diisocyanate (HDI), poly(propylene glycol) bis(2‐aminopropyl ether) with a number average molecular weight of 400 Da (D400), dimethyl sulfoxide (DMSO), *N*,*N*‐dimethylformamide (DMF), acetone (AC), dichloromethane (DCM), acetonitrile (ACN), ethyl ether (Et_2_O), methyl benzene (MB), and tetrahydrofuran (THF) were purchased from Aladdin Biochemical Technology Co., Ltd.

### Synthesis of PBD_0_ and PBD_x_

Under an atmosphere of nitrogen, PEG2000 (2 g, 1 mmol) was introduced into a 100 mL flask, which was then heated at 100 °C for 30 min. Subsequently, DMF (7 mL), DBTDL (two drops), and HDI (0.34 g, 2 mmol) were added to the mixture, and the reaction proceeded at 80 °C for 1.5 h. After this period, DB (0.141 g, 1 mmol) dissolved in DMF (5 mL) was incorporated into the existing DMF solution, and the mixture was maintained at 80 °C for 6 h to yield PBD_0_. Once the reaction mixture was allowed to cool to 60 °C, D400 (0.2 g, 0.5 mmol) in DMF (15 mL) was added, and the solution was stirred at 60 °C for an additional 30 min to produce PBD_1_. Following this protocol, variants PBD_0.25_, PBD_0.5_, and PBD_0.75_ were synthesized by adjusting the molar ratios of D400 and DB used in the feed. The solutions were poured into glass petri dishes and placed in a vacuum environment at room temperature to eliminate any bubbles. Subsequently, they were heated at 60 °C overnight, followed by drying at 80 °C for 24 h to obtain the samples.

### Determination of Solvent Resistance

For the gel fraction study, the polymer samples with a weight of *W*_1_ were soaked in THF for 24 h and then dried under vacuum. The process was repeated three times, and the samples were weighed (*W*_2_). The gel fraction *η* (%) of the polymer was calculated according to the equation:

(7) $$\eta \;=\frac{W_{2}}{W_{1}}\;\times 100$$

The solvent resistance of the polymers was determined by soaking the samples (their original mass was note as *m_0_*) into different solvents, including DMSO, DMF, AC, DCM, ACN, Et_2_O, MB, THF, and water at room temperature for 24 h. The mass of the swollen polymer was recorded as *m*_1_. The samples were then transferred to vacuum oven at 100 °C for 24 h to remove residual solvents, and the mass of the fully dried samples was noted as *m*_2_. The residual weight of the polymers was calculated according to the equation:

(8) $$Residual\;weight\;\;\left(\%\right)=\frac{m_{2}}{m_{0}}\;\times 100\%$$

### Characterizations

The molecular weights of the synthesized polymers were determined using gel permeation chromatography (GPC) on a Waters 1525 system, employing THF as the eluent at a flow rate of 1 mL min^−1^. The Fourier transform infrared (FTIR) spectra were acquired on a Bruker Tensor II spectrometer in ATR mode, spanning a range from 600 to 3800 cm^−1^. The atomic force microscopy (AFM) topographic images and AFM‐infrared (AFM‐IR) spectroscopy images at 1685 and 1645 cm^−1^ were acquired using a Bruker NanoIR2‐FS instrument. The AFM offers a spatial resolution of XY:0.2 nm and Z:0.1 nm, with a scan resolution of up to 1024 × 1024 pixels. For sample preparation, the sample was dissolved and deposited onto a silicon wafer, followed by drying at 80 °C. Proton nuclear magnetic resonance (^1^H NMR) spectra were recorded using an AVANCE III Bruker NMR spectrometer (located in Switzerland), with DMSO‐*d*_6_ serving as the solvent. The thermal stability of the polymers was evaluated using a Thermal Gravimetric Analyzer (TGA5500), with tests conducted from 30 to 550 °C at a heating rate of 10 °C min^−1^ under a nitrogen atmosphere. X‐ray photoelectron spectroscopy (XPS) examination was performed using a Thermo scientific ESCALAB Xi^+^ XPS spectrometer from Thermo Fisher using Al Kα radiation (15 kV, 20 mA), and with the C 1 s peak at 284.8 eV as an internal standard. X‐ray diffraction (XRD) patterns were obtained using a Rigaku SmartLab SE diffractometer, equipped with Cu Kα radiation (λ = 1.5418 Å), which is particularly well‐suited for high‐temperature experiments. Small‐angle X‐ray scattering (SAXS) analysis was performed on a Nanostar U SAXS instrument (Bruker, Germany), utilizing a Cu Kα X‐ray source (30 W, IµS micro‐focus source from Incoatec, Germany, λ = 0.154 nm). The periodicity (*L*) was calculated by the Bragg's law:

(9) $$L\;=\frac{2\pi }{q_{\max}}\;$$

where *q*_max_ corresponds to the peak position of the SAXS curve.

The topological freezing transition temperature (*T*_v_) was positioned between the phase transition from viscoelastic solids to viscoelastic liquids, and this transition was defined at the point where the viscosity reaches 10^12^ Pa s.^[^ ^70^ ^]^ The *T*_v_ was determined by extrapolating from stress relaxation experiments. Initially, by taking the natural logarithm of both sides of the Arrhenius equation, the equation was transformed as follows:

(10) $$\ln\left(\tau^{*}\right)\;=\frac{E_{a}}{RT}\;+ln\left(\tau_{0}\right)$$

The *T*_v_ can be determined by integrating the Arrhenius Equation (10) with the Maxwell Equation (11).

(11) $$\eta \;=\;G•\tau^{*}$$

where *η* is the viscosity (10^12^ Pa s) and *G* is the shear modulus, which is calculated based on the tensile modulus measured by DMA through the following relationship: *G* = *E′*/(2(1 + *υ*)), where *υ* is the Poisson's ratio of rubber, which is ≈0.5, and *E′* is obtained from the rubber plateau modulus in the DMA curve.

Differential scanning calorimetry (DSC) measurements were conducted using a TA Instruments DSC‐250 at a heating rate of 10 °C min^−1^ in a nitrogen atmosphere. Tensile properties were evaluated on an INSTRON 3344 universal testing machine, applying a stretching speed of 100 mm min^−1^. Rectangular samples, measuring 10 mm × 5 mm × 0.30 mm, were prepared for these tests. Dynamic mechanical properties and shape memory behavior were assessed using a DMA 850 analyzer. These analyses were performed at a consistent frequency of 1 Hz in tensile mode, spanning a temperature range from −60 to 150 °C, with a heating rate of 5 °C min^−1^. The shape memory evaluation was structured as follows:^[^ ^71^ ^]^ 1) Heating the films to 60 °C and maintaining this temperature for 5 min. 2) Applying deformation at a stress rate of 0.08 MPa min^−1^ until exceeding 0.8 MPa, followed by cooling to 0 °C and stabilizing for 10 min. 3) Releasing the applied stress and holding for an additional 10 min. 4) Raising the temperature back to 60 °C at a rate of 10 °C min^−1^, then holding for 10 min to ensure the films fully recovered. For the analysis of stress relaxation, a parallel plate probe head from an Anton Paar MCR302 rheometer, with a diameter of 25 mm, was utilized. A 1% strain was applied to measure stress relaxation, and changes in storage modulus (G′) and loss modulus (G″) at 25 and 60 °C were recorded through frequency scanning. The viscous flow temperature of the sample was measured in a range of 0 to 155 °C with a heating rate of 5 °C min^−1^ and an oscillation frequency of 1 Hz. The Shore hardness of the samples was determined using a Shore durometer (Sanliang, Japan). Additionally, the impact strength and energy absorption were measured using a cantilever impact testing machine (XJJD‐50). Photographic images and movies were recorded by a smartphone (iQOO Neo8 Pro, Shenzhen, China).

## Conflict of Interest

The authors declare no conflict of interest.

## Supporting information

Supporting Information

Supplemental Video 1

Supplemental Video 2

Supplemental Video 3

Supplemental Video 4

Supplemental Video 5

Supplemental Video 6

Supplemental Video 7

Supplemental Video 8

## Data Availability

The data that support the findings of this study are available from the corresponding author upon reasonable request.
