# Supplementary material for: Mimosa‐Inspired Body Temperature‐Responsive Shape Memory Polymer Networks: High Energy Densities and Multi‐Recyclability
Source: Adv Sci (Weinh). 2024 Aug 14;11(39):2407596. doi: 10.1002/advs.202407596 (PMC11497007; doi:10.1002/advs.202407596)
Supplement: Supplementary file 1 — Supporting Information [file ADVS-11-2407596-s004.pdf]

ADVANCED SCIENCE
Open Access

Supporting Information

for *Adv. Sci.*, DOI 10.1002/advs.202407596

Mimosa-Inspired Body Temperature-Responsive Shape Memory Polymer Networks: High Energy Densities and Multi-Recyclability

*Qingming Kong, Yu Tan, Haiyang Zhang, Tengyang Zhu, Yitan Li, Yongzheng Xing and Xu Wang**

Supporting Information

Mimosa-Inspired Body Temperature-Responsive Shape Memory Polymer Networks: High Energy Densities and Multi-Recyclability

*Qingming Kong, Yu Tan, Haiyang Zhang, Tengyang Zhu, Yitan Li, Yongzheng Xing, Xu Wang**

National Engineering Research Center for Colloidal Materials, School of Chemistry and Chemical Engineering, Shandong University, Jinan, Shandong 250100, China

*Email: wangxu@sdu.edu.cn

Descriptions for Supporting Videos

Video S1. A Mimosa-inspired shape memory polymer network curling in reaction to heat stimulation from an iron rod.

Video S2. Shape fixation of $PBD_{0.5}$ in an ice bath and its shape recovery at 60 °C.

Video S3. Shape-fixed $PBD_{0.5}$ (dimensions 33.1 mm × 5.4 mm × 0.3 mm) stretching a plastic clip (6.05 g) under infrared light, and shape-fixed $PBD_{0.5}$ (dimensions 95.2 mm × 4.2 mm × 0.3 mm) twisting a plastic clip (6.05 g) under infrared light.

Video S4. Shape-fixed $PBD_{0.5}$ (dimensions 38.9 mm × 8.6 mm × 0.3 mm) lifting a screw (2.57 g) under infrared light.

Video S5. Shape-fixed $PBD_{0.5}$ (dimensions 43.0 mm × 8.8 mm × 0.3 mm) selectively lifting a hot screw (2.57 g).

Video S6. The temporarily bent samples showing no recovery behavior at 25 °C, and exhibiting shape recovery behavior at 37 and 60 °C.

Video S7. The temporarily stretched samples showing no shape recovery behavior at 25 °C, and exhibiting shape recovery behavior at 37 and 60 °C.

Video S8. The temporarily compressed hollow cylindrical sample exhibiting shape recovery behavior at 37 °C.

Supporting Table and Figures

Table S1. Feeding molar amounts for PBD_0 and PBD_x (x = 0.25, 0.5, 0.75, and 1).

Sample	$n_{PEG2000}$ (mmol)	n_{HDI} (mmol)	n_{DB} (mmol)	$n_{D\text{-}400}$ (mmol)
PBD_0	1	2	1	0
$PBD_{0.25}$	1	2	1	0.125
$PBD_{0.5}$	1	2	1	0.250
$PBD_{0.75}$	1	2	1	0.375
PBD_1	1	2	1	0.500

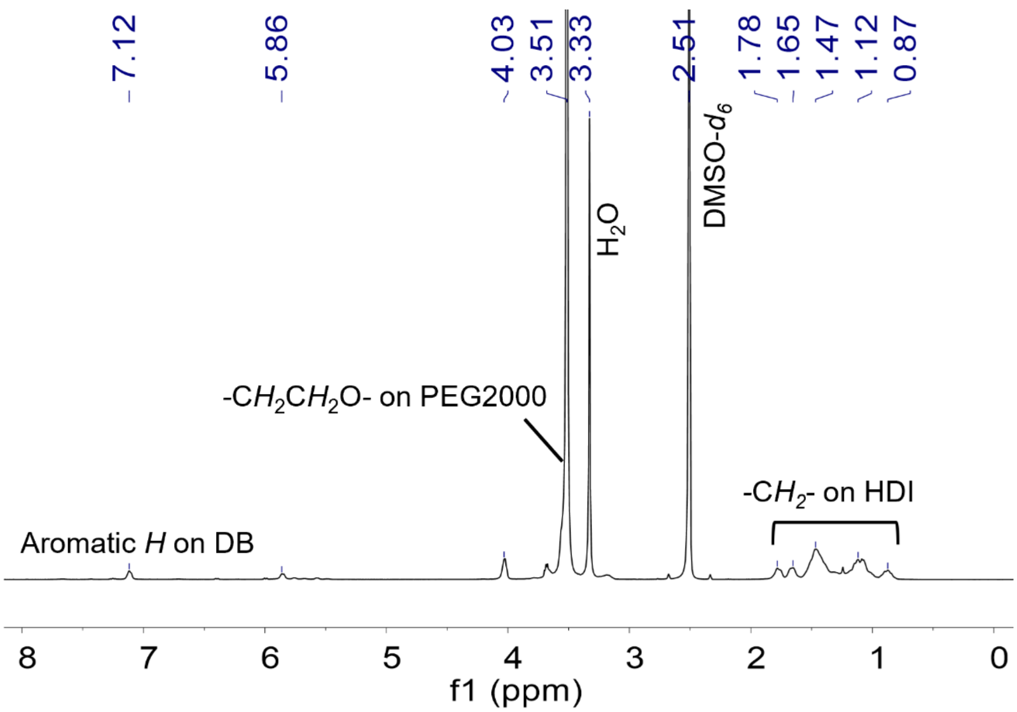

Figure S1. ^{1}H NMR spectrum of PBD_0. DMSO-d_6 as the solvent.

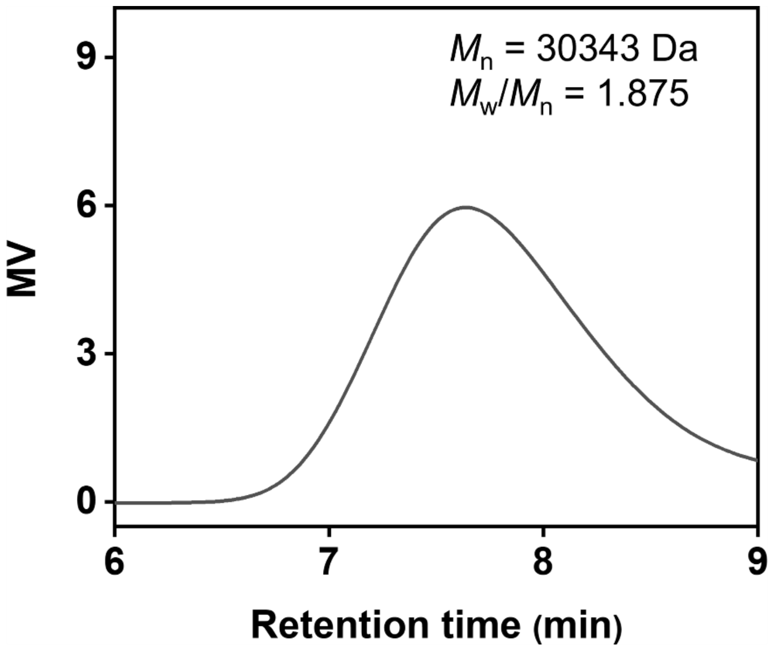

Figure S2. GPC trace of PBD_0. THF as the eluent.

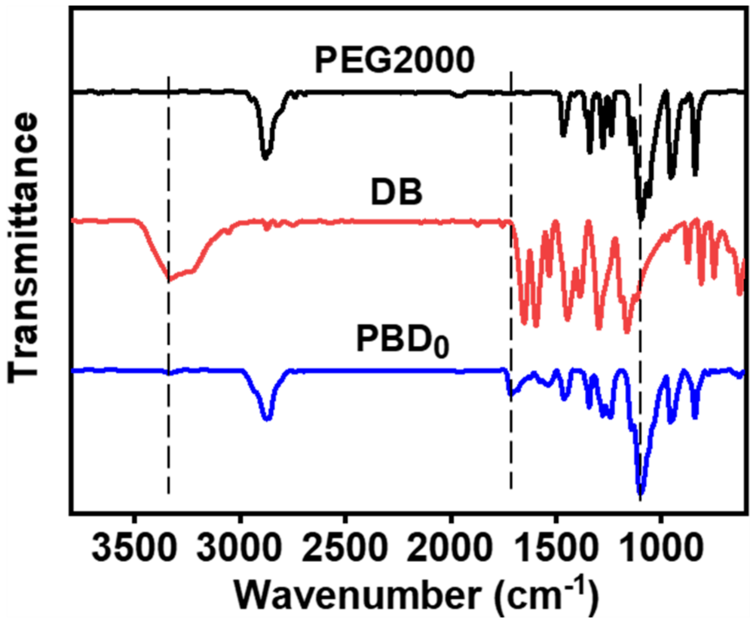

Figure S3. FTIR spectra of PEG2000, DB, and PBD_0.

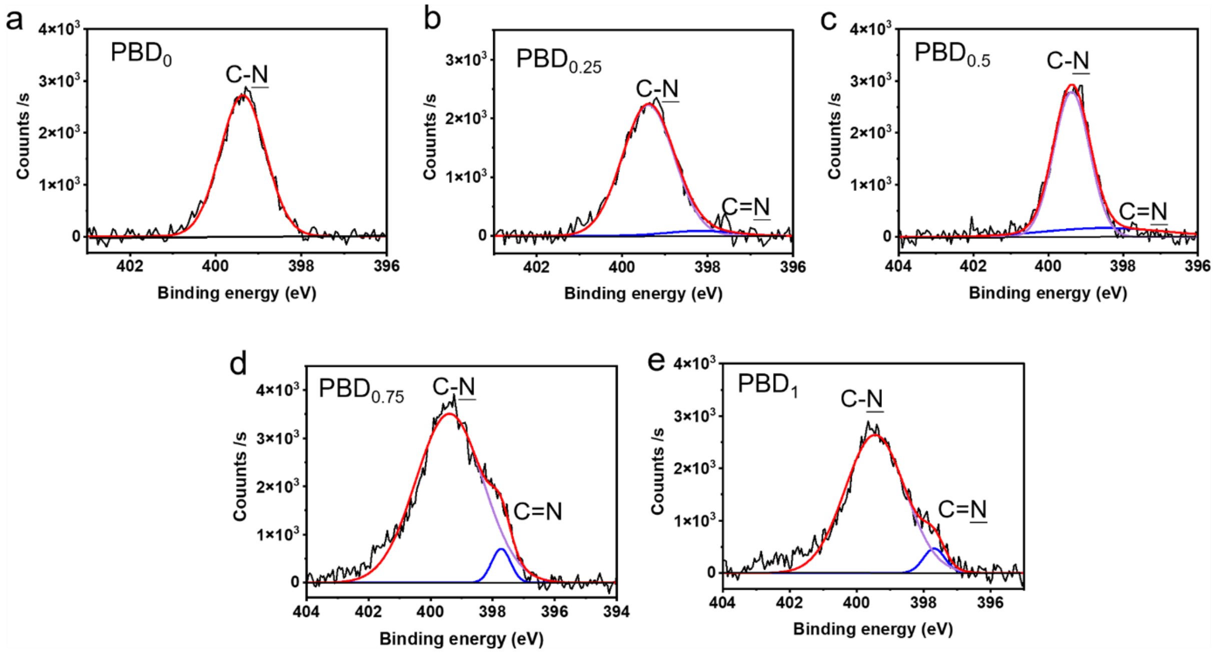

Figure S4. XPS spectra of N 1s for PBD_0 and PBD_x (x = 0.25, 0.5, 0.75, and 1).

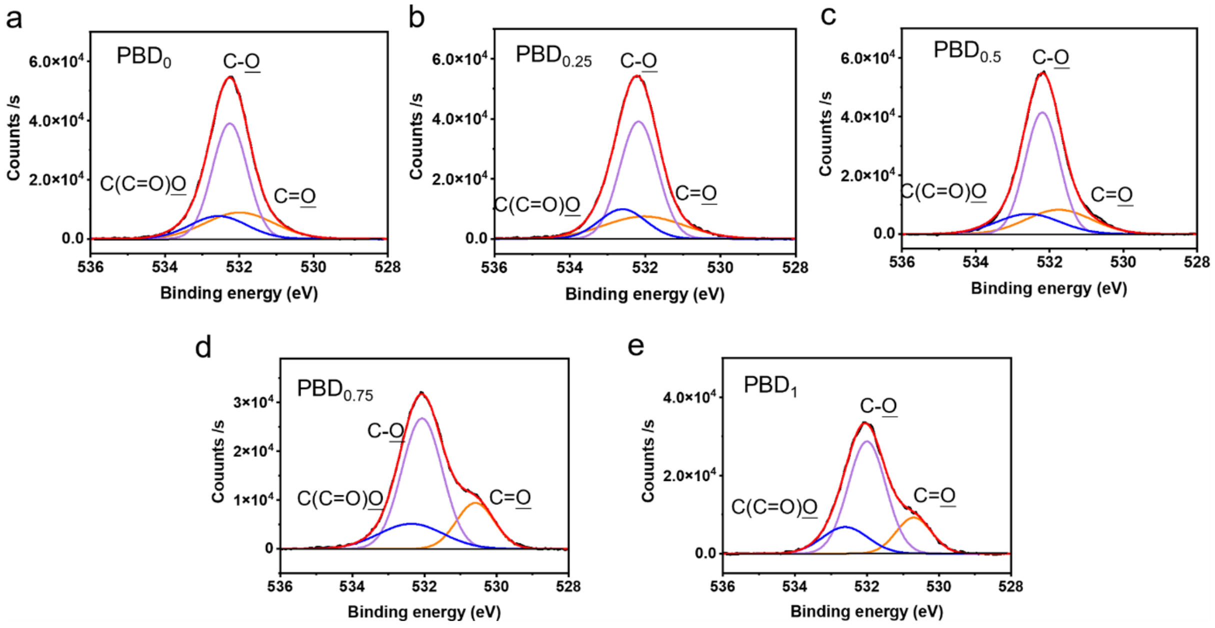

Figure S5. XPS spectra of O 1s for PBD_0 and PBD_x (x = 0.25, 0.5, 0.75, and 1).

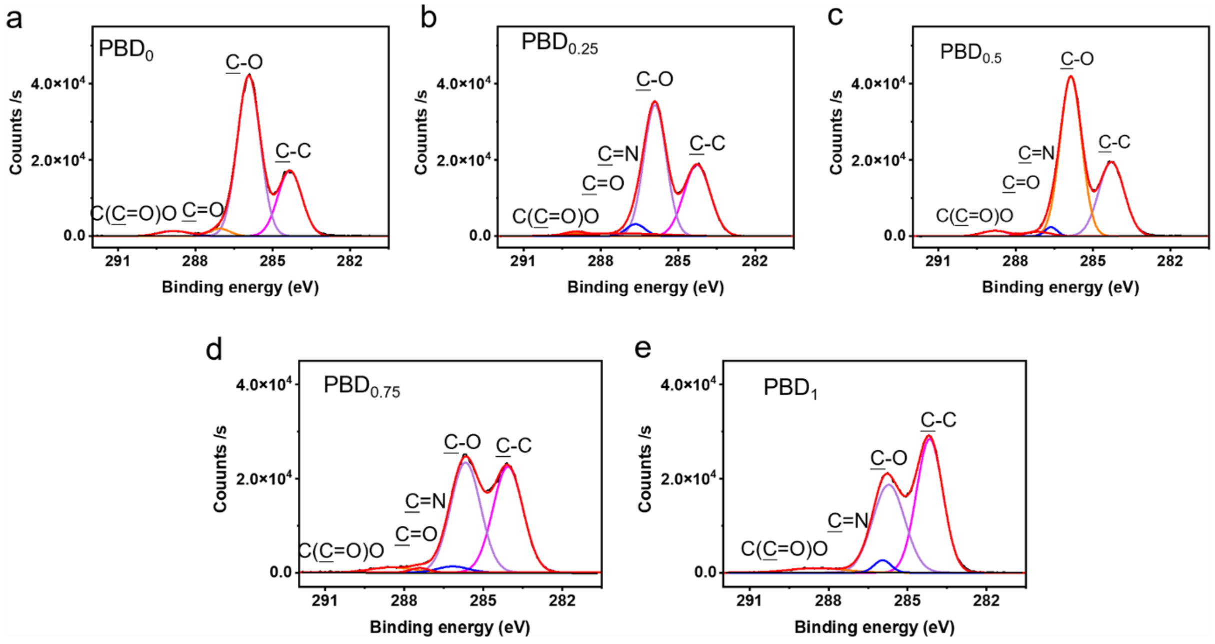

Figure S6. XPS spectra of C 1s for PBD_0 and PBD_x (x = 0.25, 0.5, 0.75, and 1).

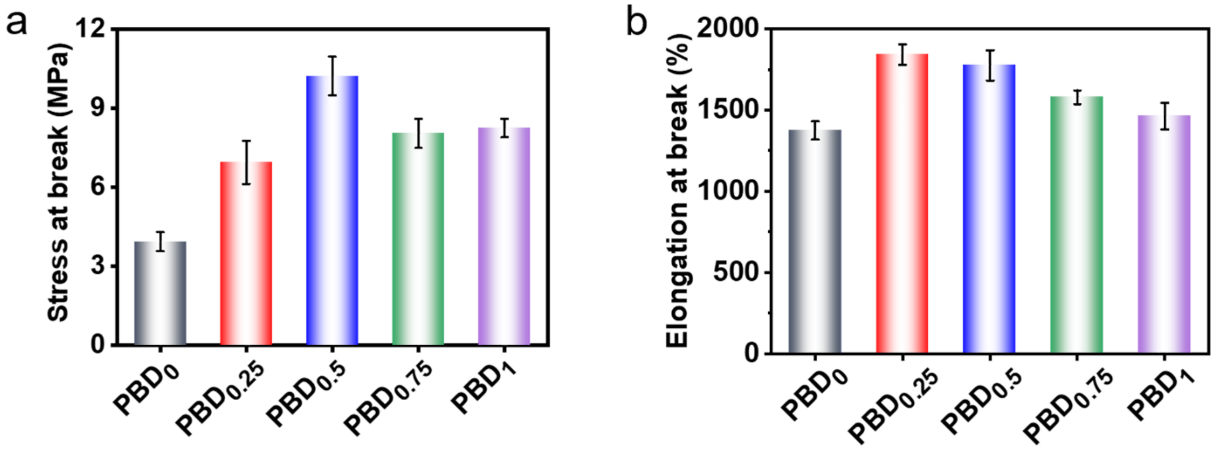

Figure S7. (a) Stress at break and (b) elongation at break of PBD_0 and PBD_x (x = 0.25, 0.5, 0.75, and 1).

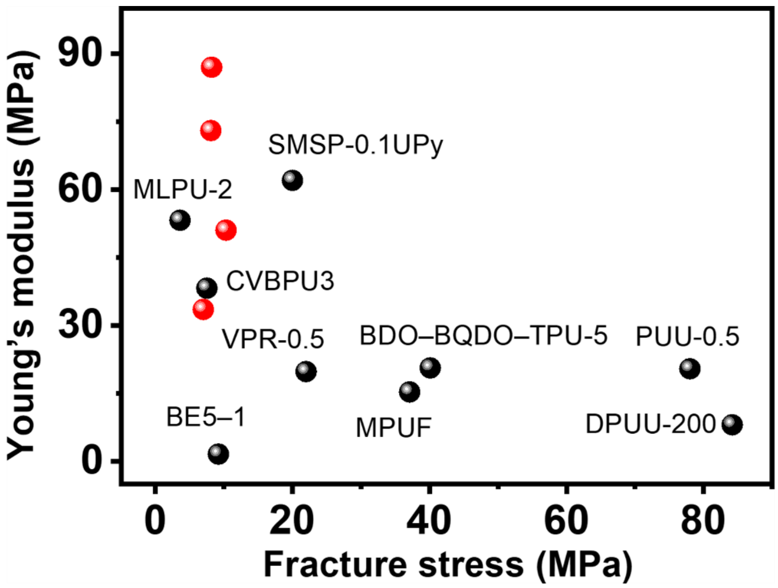

Figure S8. Comparison of fracture stress and Young's modulus between PBD_x (Red marks) and other thermosetting polymers possessing shape memory and recyclability features.[1-9]

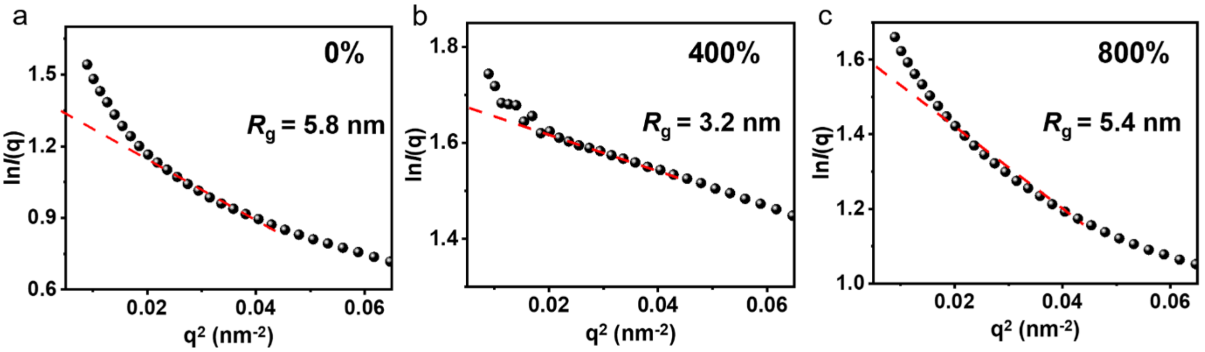

Figure S9. $\ln[I(q)] - q^2$ profiles of $PBD_{0.5}$ at stretching ratios of (a) 0%, (b) 400%, and (c) 800%.

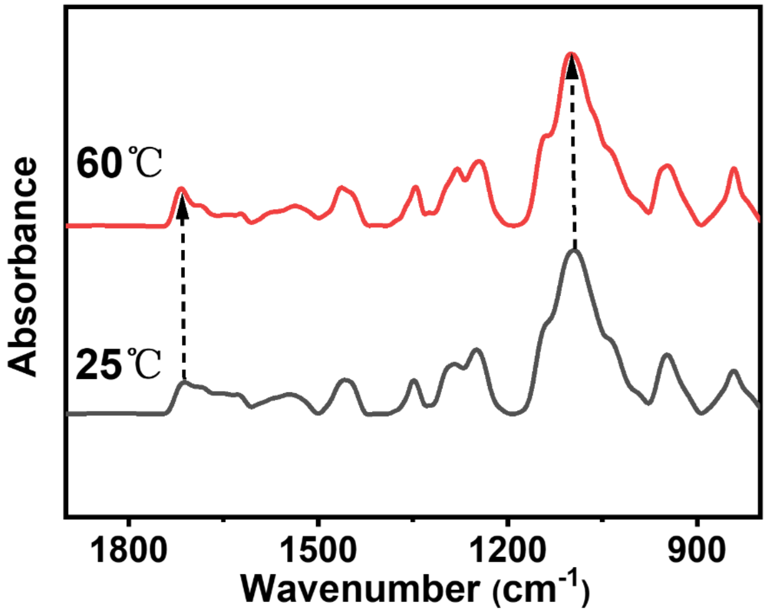

Figure S10. FTIR curves of $PBD_{0.5}$ at 25 and 60 °C.

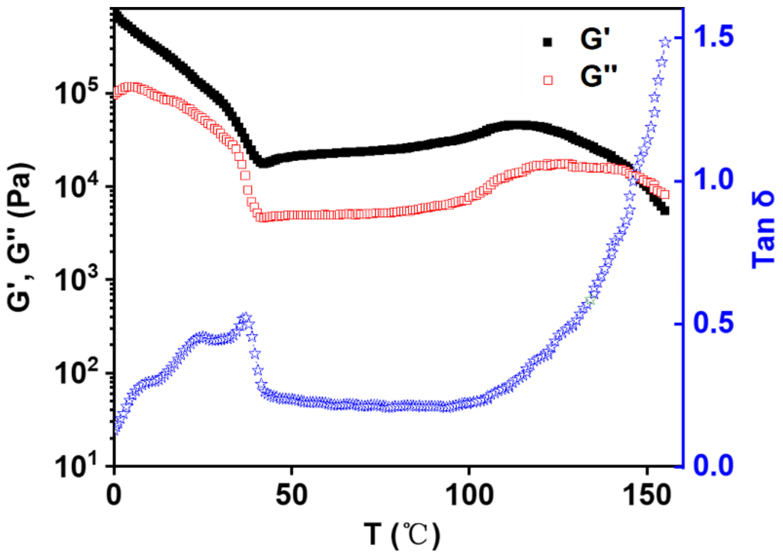

Figure S11. Dynamic rheological properties for $PBD_{0.5}$: the storage modulus G’, loss modulus G’’, and tan δ at different temperatures.

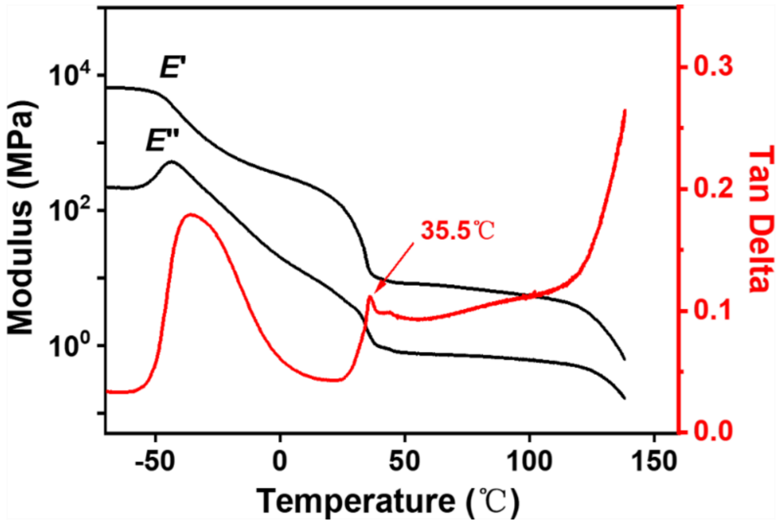

Figure S12. DMA thermogram for $PBD_{0.5}$: the storage modulus G’, loss modulus G’’, and tan δ at different temperatures.

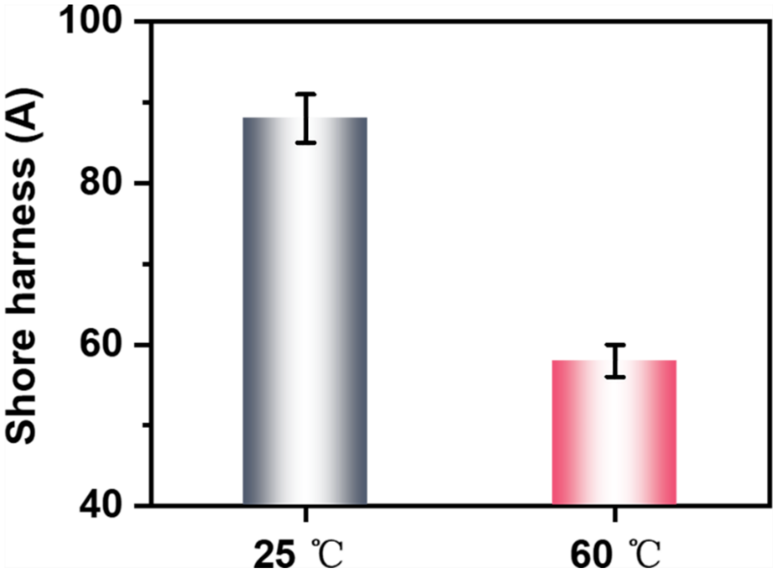

Figure S13. Shore harness of $PBD_{0.5}$ at 25 and 60 °C.

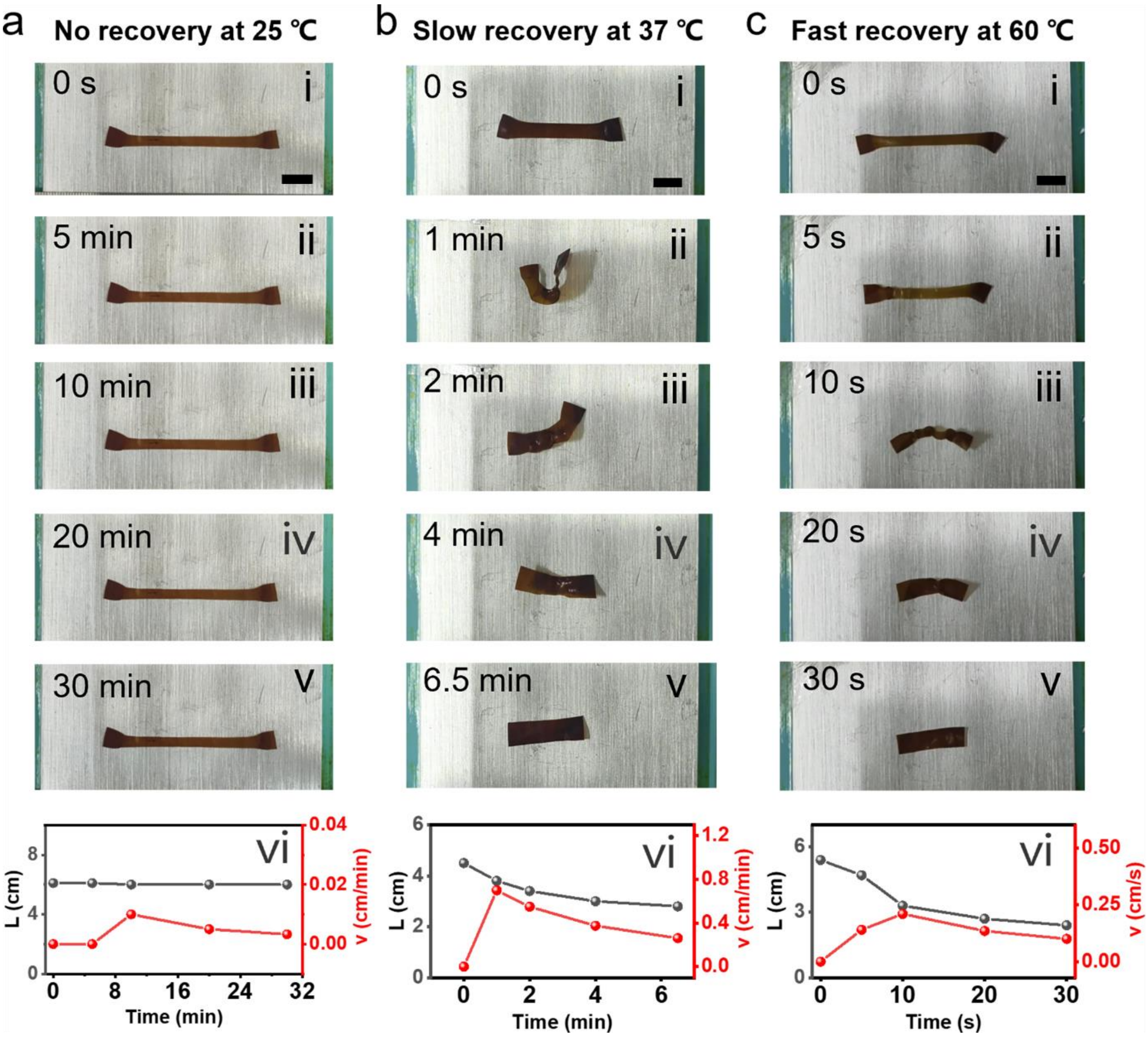

Figure S14. The shape recovery processes of $PBD_{0.5}$ at (a) 25 ℃, (b) 37 ℃, and (c) 60 ℃: (i-v) visual demonstration and (vi) the relationship between recovery rate and time.

References

[1] X. Wang, J. Xu, Y. Zhang, T. Wang, Q. Wang, S. Li, Z. Yang, X. Zhang, *Nat. Commun.* **2023**, 14, 4712.

[2] B. Zheng, T. Liu, J. Liu, Y. Cui, R. Ou, C. Guo, Z. Liu, Q. Wang, *Compos. Part.*

B-eng. **2023**, 257, 110697.

[3] X. Xu, X. Ma, M. Cui, H. Zhao, N. E. Stott, J. Zhu, N. Yan, J. Chen, *Chem. Eng. J.* **2024**, 479, 147823.

[4] S. Zhang, B. Qin, J.-F. Xu, X. Zhang, *ACS Materials Letters* **2021**, 3, 331.

[5] Z. Tang, J. Huang, B. Guo, L. Zhang, F. Liu, *Macromolecules* **2016**, 49, 1781.

[6] S. Yang, S. Wang, X. Du, Z. Du, X. Cheng, H. Wang, *Chem. Eng. J.* **2020**, 391, 123544.

[7] Y. Chen, Z. Tang, Y. Liu, S. Wu, B. Guo, *Macromolecules* **2019**, 52, 3805.

[8] X. Wang, J. Xu, X. Zhang, Z. Yang, Y. Zhang, T. Wang, Q. Wang, *Adv. Mater.* **2022**, 34, 2205763.

[9] J. Wang, X. Lin, R. Wang, Y. Lu, L. Zhang, *Adv. Funct. Mater.* **2022**, 33, 2211579.
